# Accurate classification of pain experiences using wearable electroencephalography in adolescents with and without chronic musculoskeletal pain

**DOI:** 10.3389/fpain.2022.991793

**Published:** 2022-09-27

**Authors:** Elizabeth F. Teel, Don Daniel Ocay, Stefanie Blain-Moraes, Catherine E. Ferland

**Affiliations:** ^1^ Department of Health, Kinesiology, & Applied Physiology, School of Physical and Occupational Therapy, McGill University, Montreal, QC, Canada; ^2^ Department of Experimental Surgery, McGill University, Montreal, QC, Canada; ^3^ Shriners Hospitals for Children-Canada, Montreal, QC, Canada; ^4^ Montreal General Hospital, McGill University Health Centre, Montreal, QC, Canada; ^5^ School of Physical and Occupational Therapy, McGill University, Montreal, QC, Canada; ^6^ Department of Anesthesia, McGill University, Montreal, QC, Canada; ^7^ Research Institute-McGill University Health Centre, Montreal, QC, Canada; ^8^ Alan Edwards Research Center for Pain, McGill University, Montreal, QC, Canada

**Keywords:** pain, machine learning, children, EEG, sensory testing, neuroimaging

## Abstract

**Objective:**

We assessed the potential of using EEG to detect cold thermal pain in adolescents with and without chronic musculoskeletal pain.

**Methods:**

Thirty-nine healthy controls (15.2 ± 2.1 years, 18 females) and 121 chronic pain participants (15.0 ± 2.0 years, 100 females, 85 experiencing pain ≥12-months) had 19-channel EEG recorded at rest and throughout a cold-pressor task (CPT). Permutation entropy, directed phase lag index, peak frequency, and binary graph theory features were calculated across 10-second EEG epochs (Healthy: 292 baseline / 273 CPT epochs; Pain: 1039 baseline / 755 CPT epochs). Support vector machine (SVM) and logistic regression models were trained to classify between baseline and CPT conditions separately for control and pain participants.

**Results:**

SVM models significantly distinguished between baseline and CPT conditions in chronic pain (75.2% accuracy, 95% CI: 71.4%–77.1%; *p* < 0.0001) and control (74.8% accuracy, 95% CI: 66.3%–77.6%; *p* < 0.0001) participants. Logistic regression models performed similar to the SVM (Pain: 75.8% accuracy, 95% CI: 69.5%–76.6%, *p* < 0.0001; Controls: 72.0% accuracy, 95% CI: 64.5%–78.5%, *p* < 0.0001). Permutation entropy features in the theta frequency band were the largest contributor to model accuracy for both groups.

**Conclusions:**

Our results demonstrate that subjective pain experiences can accurately be detected from electrophysiological data, and represent the first step towards the development of a point-of-care system to detect pain in the absence of self-report.

## Introduction

Chronic pain is the third leading cause of disability in adolescents aged 15 to 19 years old (1) and is estimated to affect 11%–38% of all children and adolescents (2, 3). Musculoskeletal pain is one of the most common types of chronic pain in pediatric populations and negatively affects activities of daily living and overall quality of life (4, 5). Chronic musculoskeletal pain is a dynamic condition modulated by physiological, psychological, and sociocultural factors, resulting in pain that can vary in its intensity, regularity, and predictability (6, 7). Children and adolescents with chronic pain typically see two to three physicians and wait between 12 and 24 months before being referred to a pediatric pain specialist (8–10), delaying treatments and interventions that can effectively manage pain. Ultimately, this has negative consequences in physical, academic, social, and sleep domains (5), with 32% of chronic pain patients missing ten or more days of school per year and 47% of those participating in sport quitting entirely (8). Furthermore, chronic musculoskeletal pain in adolescents has a negative financial impact on the child and their family (e.g. direct medical costs) as well as on the economy as a whole (e.g. productivity losses) (11, 12). As the pathophysiology associated with chronic pediatric pain is poorly understood, current clinical assessments are reliant upon physician interviews and observations, psychological screenings, and subjective rating scales (13).

More objective methods of diagnosing and prognosticating chronic pain are needed to improve medical management and quality of life. The need for objective biomarkers of chronic pain is particularly strong for children as they undergo changes in pain processing, perception, and response throughout development (14–16). Most clinical pain evaluations require children to self-report their pain. However, children may be less able to articulate their subjective pain experience (17, 18) or may be entirely nonverbal due to intellectual and/or developmental disabilities, which further precludes appropriate diagnosis and enrollment into effective treatment strategies. Electroencephalography (EEG) is well-positioned to fill this clinical gap, as it is a safe, reliable, and low-cost neuroimaging tool that can be translated into a point-of-care system (19, 20). Pain is also associated with complex temporal-spatial neural patterns (21); thus, the high temporal resolution of EEG systems is particularly suited for capturing information that represents the central mechanisms involved in chronic pain (22). Adults with chronic pain show altered EEG patterns compared to healthy controls, including increased frontal connectivity (23) and a shift towards increased power in higher frequency bands (24). Other studies investigate the neurophysiological response of healthy adults exposed to a tonic pain stimulus (e.g. the cold pressor task (CPT)), as this is thought to better mimic true pain responses compared to phasic pain models (25, 26). Several EEG features, including spectral power derived from both fast Fourier (27, 28) and continuous wavelet (29) transformations, functional connectivity (27), and time-frequency outcomes (30, 31), differentiate between resting (e.g. no pain) and tonic cold pain conditions in heathy participants, with frontal theta rhythms appearing particularly sensitive to cold pain (32).

Growing evidence suggests that EEG features can detect both acute and chronic pain, but several factors preclude the translation of these findings to pediatric participants. 1) Most of the available studies have been conducted exclusively with adult participants. As there are distinct patterns of brain activation between adults and children in both healthy (32) and chronic pain participants (33), it is likely that these findings do not translate to adolescents. 2) Quantitative sensory testing modalities, such as the CPT, are used in chronic pain patients to differentiate between pain mechanisms, quality, and/or neurophysiological correlates, which can help guide and personalize treatment (34, 35). To date, the literature evaluating EEG changes during a CPT has focused on healthy participants. As individuals with chronic pain have altered EEG patterns at rest (36), these changes may not translate to children with chronic pain. 3) Finally, movement artifacts in EEG are a common problem in pain research (29, 37), as pain can cause changes in facial expressions (38) and gross motor movement. Furthermore, most clinical pain assessments require behavioral ratings of pain (e.g. verbal indication, hand signal, or by moving a sliding scale) (27–29), which may intrinsically generate movement artifact in the EEG signal.

We address all three of the aforementioned limiting factors in the current study, with the long-term goal of developing an effective point-of-care system for the detection of pediatric pain. The objective of this study was to assess the potential of using EEG-derived features to detect cold thermal pain in adolescents with and without chronic musculoskeletal pain using machine learning (ML) and inferential statistics approaches. We hypothesized that EEG features would accurately classify resting state and tonic cold conditions regardless of whether ML or inferential approaches were modeled. Furthermore, we hypothesized that adolescents with chronic musculoskeletal pain would have unique neurophysiological responses to the CPT compared to healthy children.

## Materials and methods

This is a sub-study of a larger project investigating clinical pain and EEG outcomes at rest and during thermal quantitative sensory testing (QST) in adolescents with and without chronic musculoskeletal pain. The methods of the larger study are described in Ocay et al. 2022 (In Review). The current study focused on comparing the classification accuracy of EEG features in healthy and chronic pain participants during no-pain (resting baseline) and pain (tonic cold) conditions using ML algorithms and inferential statistics approaches. All participants (and their parent, if younger than 14 years old at the time of enrollment) provided written informed consent. This study was approved by the Research Ethics Board at McGill University (A09-M17–17B).

### Participants

Adolescents with chronic musculoskeletal pain were recruited from the Edwards Family Interdisciplinary Center for Complex Pain at the Montreal Children's Hospital, as well as spine and orthopedic outpatient clinics at the Shriners Hospitals for Children-Canada between October 2018 and June 2021. Participants with chronic musculoskeletal pain were included if they were between 10 and 18 years old, reported musculoskeletal pain once or more per week for at least the preceding three months, and could understand and complete the clinical outcomes associated with the larger study. Age-matched healthy controls with no history of musculoskeletal pain within the last three months were recruited through word of mouth and recruitment flyers placed in local magazines and on social media. Participants were excluded if they were unable to communicate in English or French, had diagnosed development delays, or had severe systemic disease resulting in functional limitations.

### Thermal experimental pain condition

All participants underwent thermal QST previously described by our research group (39, 40). Only the cold thermal condition was used in this study. Each participant underwent a CPT, immersing their left forearm in cold water (12 °C) for 2 min. Participants verbally rated their pain every 15 s throughout the CPT, using a numerical rating scale ranging between 0 (no pain) and 10 (worst pain imaginable). An average pain intensity score of 10 (maximum value) was given if a participant was unable to keep their forearm submerged for the full 2-minute protocol.

### Electroencephalography (EEG) methods

#### EEG recording and processing

A dry EEG headset (DSI-24, Wearable Sensing) was used to record electrical activity during an eyes-open resting state (i.e. no-pain) assessment and throughout the CPT. To investigate the effect of motor movement on EEG features, a subset of participants arbitrarily moved a computerized visual analogue scale (used in the tonic heat condition of the larger study) during the baseline assessment while the remaining participants completed the baseline with no voluntary motor movements.

EEG data was recorded at 300 Hz, referenced to the Pz electrode, and impedance was kept below 1 MΩ in accordance with manufacturer recommendations. Following data collection, the EEG signal was loaded into EEGlab (41), where it was preprocessed as follows: 1) data were bandpass filtered between 0.1–50 Hz; 2) EEG was re-referenced from Pz to A1 and A2 (ear lobe) electrodes; 3) bad channels, if present, were removed from the dataset; 4) an Independent Component Analysis was conducted to remove repetitive noise in the signal (eye blinks, eye movements, etc.); and 5) any remaining bad segments of EEG data were manually removed following a visual inspection of the signal and segments were rejoined (i.e. concatenated) at the boundary. Missing values resulting from the removal of noisy channels were imputed using the mean value from all existing data points for a given feature (e.g. column average).

#### EEG feature extraction

The preprocessed EEG was segmented into 10 s, non-overlapping epochs and analyzed in Matlab using custom scripts. All features described below were calculated at four frequency bands: 1) delta (1–4 Hz), 2) theta (4–8 Hz), 3) alpha (8–13 Hz), and 4) beta (13–30 Hz). Seven classes of EEG features were calculated for each 10-second epoch: spectral power, peak frequency, permutation entropy (PE), weighted phase lag index (wPLI), directed phase lag index (dPLI), and graph theory features (path length, clustering coefficient, small-world architecture, modularity, and node strength).

Spectral features, which assess the power of oscillatory components of the EEG signal, were computed using the spectopo function from EEGlab. Spectral power outcomes were generated using the multitaper method, with number of tapers K = 3 and a time-bandwidth product NW = 2. Peak frequency (Hz) was calculated as that with the largest power amplitude within each frequency band. Spectral power outcomes were generated for each channel, while peak frequency was calculated across all channels.

Permutation entropy assesses the information content of the EEG signal. The continuous EEG signal was mapped onto a symbolic sequence of motifs according to shape (slope, peaks, and troughs). This mapping is specified through an embedding dimension (number of samples included in each motif) and time lag (number of samples spanned by each section of the motif) (42). We selected an embedding dimension of 5 and time lag of 4 to ensure a sufficient deployment of the trajectories for the beta frequency band (43). Higher (approaching 1) normalized PE value indicates that the EEG signal contains predominately higher frequencies, while lower (approaching 0) normalized PE value implies that the EEG signal contains predominately lower frequencies. PE features were calculated for each channel.

Weighted phase lag index (wPLI) was used to calculate the degree of functional coupling across all electrode pairs, as this phase-based functional connectivity metric is minimally susceptible to volume conduction (44). For each channel, instantaneous phase of the EEG was calculated using a Hilbert transform. Then, the phase difference between each pair of electrodes was calculated and weighted by the magnitude of the imaginary component of the cross-spectrum. The wPLI ranges between 0 (i.e. no coupling) and 1 (i.e. strong coupling).

To determine the direction of the phase lead/lag relationships between electrode pairs, the dPLI was calculated (45). The dPLI ranges between 0 and 1: 0.5 ≤ dPLI ≤ 1 indicates that electrode 1 leads electrode 2, 0 ≤ dPL I≤ 0.5 indicates that electrode 2 leads electrode 1, and dPLI = 0.5 indicates no consistent phase lead/lag relationship between electrodes. Both wPLI and dPLI yield a 19 × 19 functional connectivity matrix, with each cell in the matrix representing the strength of the functional connection between a pair of electrodes. To generate the average connectivity value per electrode, we calculated the mean across each row of the matrix.

Graph theory analyses, which synthesize functional connectivity into graph representations to evaluate network-based outcomes, were performed using both weighted and binary networks. To create weighted networks, graphs were derived from raw wPLI matrices (e.g. continuous values between 0 and 1). To create binary networks, the wPLI matrices were transformed such that functional connections above a given threshold were set to 1 and the remaining connections were set to 0. The threshold was set to the percentage of connections required to maintain a minimally spanning graph for the baseline EEG; separate thresholds were calculated for each participant at each frequency band. All graphs (e.g. weighted and binary) were normalized against 100 random networks that shuffled the network's edges while preserving the degree and strength distributions. From the weighted and binary networks, we calculated graph theoretical properties of path length (46), clustering coefficient (47, 48), small-world architecture (47), modularity (49), and node strength. The code underlying the graph theory analysis is found in the Brain Connectivity Toolbox (50).

Ultimately, a total of 380 EEG features were calculated: 76 (19 channels  × 4 frequency bands) spectral power features, 4 (1 outcome  × 4 frequency bands) peak frequency features, 76 (19 channels  × 4 frequency bands) PE features, 76 (19 channels  × 4 frequency bands) wPLI features, 76 (19 channels  × 4 frequency bands) dPLI features, 36 (9 features  × 4 frequency bands) binary graph theory features, and 36 (9 features  × 4 frequency bands) weighted graph theory features.

### Machine learning methods

We implemented a ML framework for an epoch-by-epoch binary classification of no-pain (baseline) vs. pain (CPT). As our prior work found significant differences in EEG features between healthy controls and chronic pain participants (Ocay et al. 2022, In Review), models were run separately for each group. As the range of possible values of the EEG features varied widely, a standard scaler normalization was applied to ensure equal weighting across all EEG features in the machine learning analysis. Across all groups and conditions, an observation space (i.e. total number of 10-second epochs on which EEG features were extracted) of *n* = 2,359 (Healthy: no-pain = 292 epochs, pain = 273 epochs; Chronic Pain: no-pain = 1039 epochs, pain = 755 epochs) was formed. The entire analysis pipeline was developed and implemented using scikit-learn (51).

#### Model selection

We evaluated multiple binary classification algorithms to identify the optimal ML approach to separate no-pain and pain conditions. These models included linear-discriminant analysis (LDA), support vector machines (SVM), and decision trees. To compare the ML approaches to more traditional inferential statistics methods, we also evaluated logistic regression models. For the SVM and logistic regression models, we conducted a hyperparameter sweep (0 to 1 by 0.1 steps, 2, 5, 10) of the regularization parameter C. Both linear and radial basis function kernels were also evaluated for the SVMs, while the gini and entropy criterion hyperparameters were tested for the decision trees. The LDA was conducted using only default hyperparameters in scikit-learn.

EEG epochs derived from a single participant are highly correlated; to ensure independence between the training and test sets, a leave-one-subject-out (LOSO) cross-validation scheme was used. The overall accuracy reported for each model was calculated as the average accuracy over all LOSO repetitions. The ML and logistic regression model (and their associated hyperparameters) with the highest classification accuracy were selected for subsequent analyses.

Feature importance of the final model was evaluated using the logistic regression model weights observed during the final model performance on the validation set, as model coefficients cannot be generated when using a radial basis function kernel for SVM models. As all features were normalized using a standard scalar prior to the machine learning analysis, no additional scaling of the model coefficients was performed.

### Statistical analysis

#### Demographic information and effect of movement on EEG features

Independent sample *t*-tests (continuous data) or chi-square analyses (categorical data) compared demographic characteristics between chronic pain and heathy participants, with significance set at *p* = 0.05. Prior to the machine learning analysis, independent sample *t*-test were also conducted to evaluate differences in no-pain EEG features between those participants who performed small, arbitrary motor movements at rest compared to those completing the baseline fully at rest (i.e. no voluntary motor movements). Each EEG feature (power, peak power, etc.) was analyzed separately; within features, all channels and frequency bands were combined. Bonferroni corrections were applied based on the 7 classes of EEG features analyzed, setting the significance level at *p* = 0.007. Any EEG features determined to be significantly affected (*p *≤ 0.007) by motor movements were removed from the dataset prior to the ML analysis. To provide further evidence regarding the effect of motor activity on baseline EEG features, Cohen's d values are reported and interpreted as negligible (*d* < 0.2), small (0.2 ≤ *d* < 0.5), medium (0.5 ≤ *d* < 0.8), and large (*d* ≥ 0.8) effect sizes.

#### Machine learning model performance

The full dataset was randomly split into an 80:20 train/test and validation dataset, implemented with the test_train_split function in scikit-learn. Model selection and initial model performance metrics were performed on the train/test set using the LOSO cross-validation scheme described above. Once the best models and their associated hyperparameters were selected, final model performance was determined using the validation set. To determine if particular EEG features contributed more strongly to the classification accuracy of the model, the final models were run on the full dataset (all EEG features combined) and on each EEG feature individually (PE features only, graph theory features only, etc.).

Permutation testing assessed the statistical significance of model performance. Model accuracy was calculated on 10,000 iterations through a permutated dataset, creating a null distribution of random accuracy. Model performance was deemed to be statistically significant if the accuracy of the true model (i.e. non-permutated labels) was greater than the permutated model at a level of *p* = 0.01. To establish 95% confidence intervals, a bootstrapping procedure was performed. Bootstrapped datasets were created by sampling from the original dataset with replacement. Similar to the permutation testing, model accuracy was calculated on 10,000 bootstrapped datasets to create a null distribution of classifier performance. The 95% confidence intervals were created using the 2.5 and 97.5 percentiles of the bootstrapped distribution. To compare between group (healthy vs. chronic pain) and model type (ML or inferential statistics), models were deemed to have significantly different classification accuracy if their confidence intervals did not overlap.

## Results

**Figure 1:**
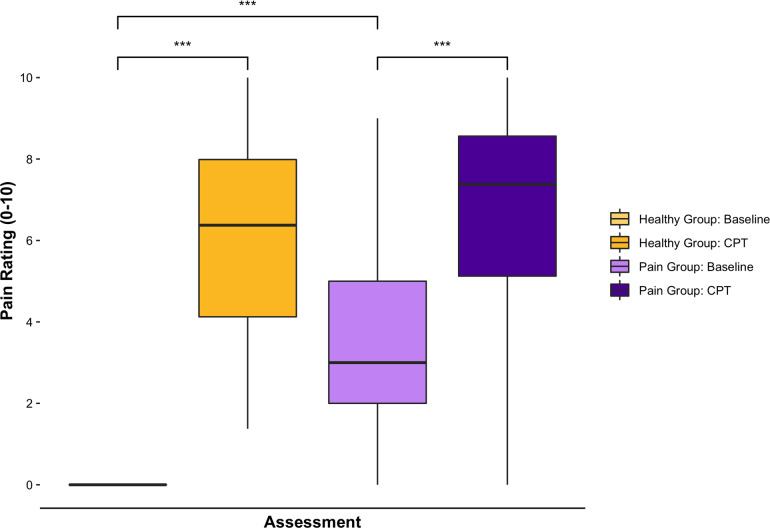
Subjective pain ratings before and after the CPT. Bar charts displaying self-reported pain scores (numeric rating scale 0–10) at baseline and during the CPT (average score throughout the condition). All healthy participants reported no pain (0) at baseline. Both healthy and chronic pain participants had significantly higher pain scores during the CPT compared to baseline. Chronic pain participants had significantly higher scores than healthy participants at baseline, but average pain scores throughout the CPT were not significantly different between groups. ***denotes significance at *p* < 0.001 level.

A total of 160 adolescents (39 controls, 121 pain) completed the study. Both groups were similar in age (Control: 15.2 ± 2.1, Pain: 15.0 ± 2.0, *t* = 0.49, *p* = 0.62), but the chronic pain group had a significantly higher proportion of females (Control: *n* = 18 (46.2%), Pain: *n* = 100 (82.6%), *χ*2(1) = 20.3, *p* < 0.0001). The majority of participants in the chronic musculoskeletal pain group reported pain for more than 12 months (*n* = 85, 70.3%), while 22 (18.2%) reported pain between 6 and 12 months, and 14 (11.6%) report pain for less than 6 months. Most pain participants reported their pain frequency as at least once per day (*n* = 97, 80.2%) or every other day (*n* = 16, 13.2%), with the duration of each episode ranging from a few minutes (*n* = 17, 11.9%) to constant (*n* = 95, 66.4%). At baseline, no healthy participants reported any pain, while chronic pain participants reported a pain score of 3.4 ± 2.4 (*t *= −15.5, *p* < 0.0001). The average pain score recorded throughout the CPT was significantly higher than baseline for both healthy (6.1 ± 2.3, *t *= −16.6, *p* < 0.001) and chronic pain (6.7 ± 2.4, *t *= −10.4, *p* < 0.001) participants; however, there were no differences in the average pain rating between healthy and chronic pain groups throughout the CPT (*t *= −1.27, *p* = 0.21, Figure 1). Approximately half (*n* = 72, 45%) of participants performed arbitrary motor movements using the computerized visual analogue scale at baseline to examine the influence of motor movements on the EEG features.

### Voluntarily motor movement affects a subset of EEG features

Spectral power (*p* < 0.001, Cohen's *d* = 0.84), wPLI (*p* < 0.001, Cohen's *d* = 0.40), and weighted graph theory outcomes (clustering coefficient: *p* = 0.002, Cohen's *d* = 0.19; small-world architecture: *p* = 0.006, Cohen's *d* = 0.17; node strength: *p* < 0.001, Cohen's *d* = 0.38) significantly differed between participants who did and did not complete small, arbitrary motor movements during the no-pain EEG assessments. All of the remaining EEG variables were not significantly different between groups and had Cohen's d values <0.20 (negligible effect size), indicating that these outcomes were not affected by voluntary motor activity. Spectral power, wPLI, and weighted graph theory outcomes were removed from the dataset, with peak frequency, dPLI, PE, and binary graph theory features retained for the no-pain vs. pain classification analysis.

### EEG classifies pain with 75% accuracy in chronic musculoskeletal pain participants

#### Model performance on the test/train sets

The SVM with radial basis function kernel and *C* = 0.9 yielded the highest classification accuracy of all ML models, while an L1 penalty and *C* = 0.1 produced the best performance for the logistic regression models. The SVM model distinguished between no-pain and pain conditions with 75.6% accuracy (95% CI: 71.9%–77.6%; *p* < 0.0001). The logistic regression distinguished between no-pain and pain conditions with 74.8% accuracy (95% CI: 71.1%–77.6%; *p* < 0.0001). No performance differences were observed between the SVM and logistic models, as evidenced by their overlapping confidence intervals. The performance of individual features is found in the Table 1. In both the SVM and logistic regression models, dPLI, PE, and peak frequency features significantly classified between no-pain and pain conditions as independent features.

**Table 1:** Test/train and validation set accuracies for the radial SVM and logistic regression models presented for the chronic pain and healthy controls groups.

Model Type	Features	Chronic Pain Participants				Healthy Controls			
		Test/Train Set		Validation Set		Test/Train Set		Validation Set	
		Accuracy (%)	*P*	Accuracy (%)	*P*	Accuracy (%)	*P*	Accuracy (%)	*P*
SVM	All	75.59 (71.92, 77.58)	<0.001	75.20 (71.39, 77.11)	<0.001	74.43 (68.22, 78.39)	<0.001	74.77 (66.36, 77.57)	<0.001
	dPLI Only	56.57 (54.83, 62.58)	0.002	59.67 (54.50, 61.03)	<0.001	53.04 (43.64, 59.84)	0.17	43.93 (37.38, 53.27)	0.88
	Graph Only	57.68 (51.72, 60.26)	0.09	53.68 (47.96, 55.86)	0.84	55.63 (46.22, 61.93)	0.11	59.81 (49.53, 62.62)	0.02
	PE Only	74.36 (70.64, 76.73)	<0.001	72.75 (67.03, 74.11)	<0.001	70.56 (65.99, 77.88)	<0.001	71.96 (66.36, 78.50)	0.009
	Peak Only	61.26 (57.04, 64.64)	<0.001	59.13 (55.86, 59.95)	<0.001	55.30 (48.61, 65.08)	0.008	55.14 (47.66, 58.88)	0.07
Logistic Regression	All	74.84 (71.10, 77.61)	<0.001	75.75 (69.48, 76.57)	<0.001	72.76 (65.88, 78.91)	<0.001	71.96 (64.49, 78.50)	<0.001
	dPLI Only	58.44 (53.02, 61.20)	0.007	61.58 (52.86, 61.59)	<0.001	51.78 (43.68, 60.09)	0.26	50.47 (38.32, 57.94)	0.45
	Graph Only	57.30 (52.80, 60.63)	0.05	59.95 (53.13, 60.49)	<0.001	55.09 (45.33, 62.13)	0.03	59.81 (49.53, 65.42)	0.02
	PE Only	74.67 (70.62, 76.52)	<0.001	75.20 (70.02, 76.02)	<0.001	73.44 (68.02, 80.04)	<0.001	74.77 (66.36, 78.50)	0.002
	Peak Only	60.17 (57.48, 63.86)	<0.001	58.58 (56.40, 60.49)	<0.001	55.23 (46.20, 62.08)	0.13	54.21 (48.60, 58.88)	0.006

**Note**: 95% Confidence intervals are presented below the point accuracy in parentheses. dPLI = directed phase lag index, Graph = binary graph theory, PE = permutation entropy, and Peak = peak frequency.

#### Final model performance on the validation set

Overall model performance was largely replicated on the validation set. Final model accuracy was 75.2% (95% CI: 71.4%–77.1%, *p* < 0.0001) for the SVM and 75.8% (95% CI: 69.5%–76.6%, *p* < 0.0001) for the logistic regression. In both instances, the model accuracy was within 1% point of the observed performance on the test/train set. For the SVM model, PE, dPLI, and peak frequency features could all significantly classify between no-pain and pain conditions as stand-alone features; only the binary graph theory features (53.7% accuracy, 95% CI: 48.0%–55.9%, *p* = 0.84) were unable to independently classify between the conditions. For the logistic regression model, all features accurately classified between no-pain and pain conditions (range: 58.6%–75.2% accuracy) at a level greater than chance (Table 1).

### EEG classifies pain with 72%–74% accuracy in healthy participants

#### Model performance on the test/train sets

The highest performing SVM model in the healthy control group used a radial basis function kernel and *C* = 0.8, while the best performing logistic regression model was identical to the chronic pain participants (L1 penalty and *C* = 0.1). Model performance in the healthy control group was nearly identical to the chronic pain group, with the SVM classifying between no-pain and pain conditions with 74.4% accuracy (95% CI: 68.2%–78.4%, *p* < 0.0001) and the logistic regression classifying with 72.8% accuracy (95% CI: 65.9%–78.9%, *p* < 0.0001). PE features significantly classified between no-pain and pain conditions for both SVM and logistic regression models (SVM: 70.6% accuracy, 95% CI: 66.0%–77.9%, *p* < 0.0001; Logistic: 73.4% accuracy, 95% CI: 68.0%–80.0%, *p* < 0.0001, Table 1), while peak frequency features were significant stand-alone classifiers in the SVM model only (55.3% accuracy, 95% CI: 48.6%–65.1%, *p* = 0.008).

#### Final model performance on the validation set

Overall model performance was replicated for healthy participants using the validation set, with classification accuracy equivalent to test/train model performance as evidenced by the overlapping confidence intervals. The overall SVM model with all EEG features included classified between no-pain and pain conditions with 74.8% accuracy (95% CI: 66.3%–77.6%, *p* = 0.0007), while the logistic regression performed at 72.0% accuracy (95% CI: 64.5%–78.5%, *p* = 0.0003). For the SVM model, only PE features could independently classify between no-pain and pain at a significant level (72.0% accuracy, 95% CI: 66.3%–78.5%, *p* = 0.009). For the logistic regression model, both PE (74.8% accuracy, 95% CI: 66.3%–78.5%, *p* = 0.002) and peak frequency (54.2% accuracy, 95% CI: 48.6%–58.9%, *p* = 0.006) features could classify at a level above chance (Table 1).

### Theta permutation entropy is most predictive of pain experience, with different regions of importance between groups

**Figure 2:**
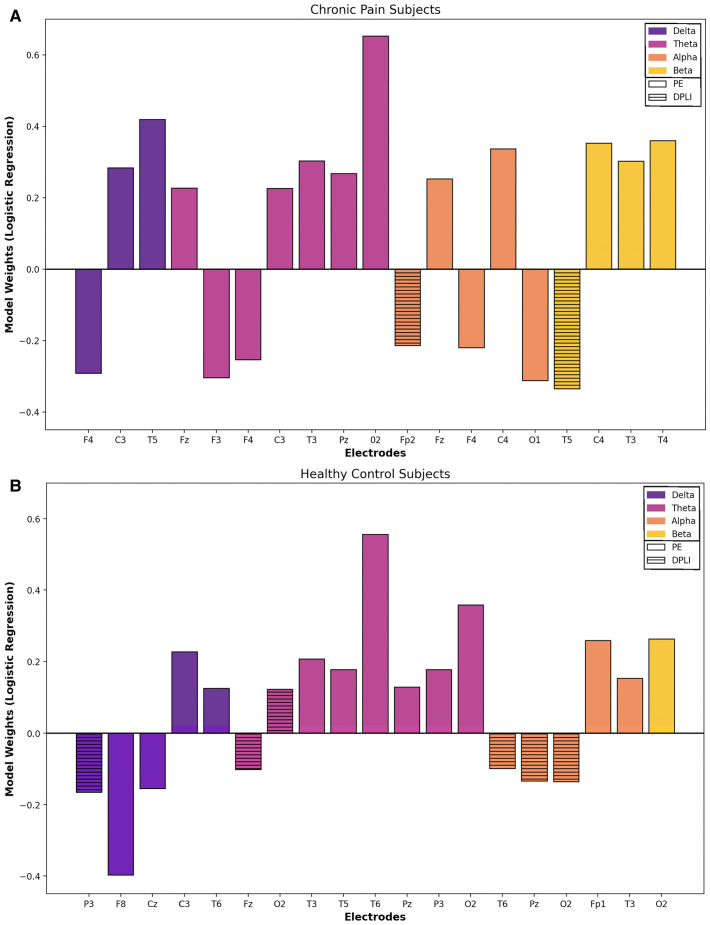
Baseline vs. CPT: feature importance. Most important features (top 10%) for classifying between pain and no pain conditions based on logistic regression model weights for chronic pain (**A**) and healthy (**B**) participants. **Note**: Model coefficients cannot be generated when using a radial basis function kernel for SVM models; thus, overall model feature importance was only explored using the logistic regression models. Solid boxes represent permutation entropy features, while stripped boxes indicate directed phase lag index features. No graph theory or peak frequency features were in the top 10% based on model coefficients. Positive model weights are predictive of tonic cold pain conditions, while negative model weights are predictive of no-pain conditions.

The most important features (i.e. the top 10% based on the absolute value of the model coefficient) for chronic pain and healthy participants are visualized in Figure 2. PE features were the most important feature for both groups, aligning with the individual EEG feature results presented above. PE features accounted for 89.5% (*n* = 17) and 68.4% (*n* = 13) of the most important features for chronic pain and healthy participants, respectively. The remaining features were derived from the dPLI analysis, with no peak frequency or binary graph theory features appearing in the top 10% of the logistic regression model coefficients. For both groups, the majority of features were in the theta (Pain: *n* = 7, 36.8%; Control: *n* = 8, 42.1%) and alpha (both groups: *n* = 5, 26.3%) frequency bands. Models in chronic pain participants place more importance on features in frontal (Pain: *n* = 7, Controls: *n* = 3) and central (Pain: *n* = 4; Controls: *n* = 2) regions; conversely, feature importance in healthy participants was predominantly in parietal (Pain: *n* = 1; Controls: *n* = 4) and occipital (Pain: *n* = 1; Controls: *n* = 4) areas.

**Figure 3:**
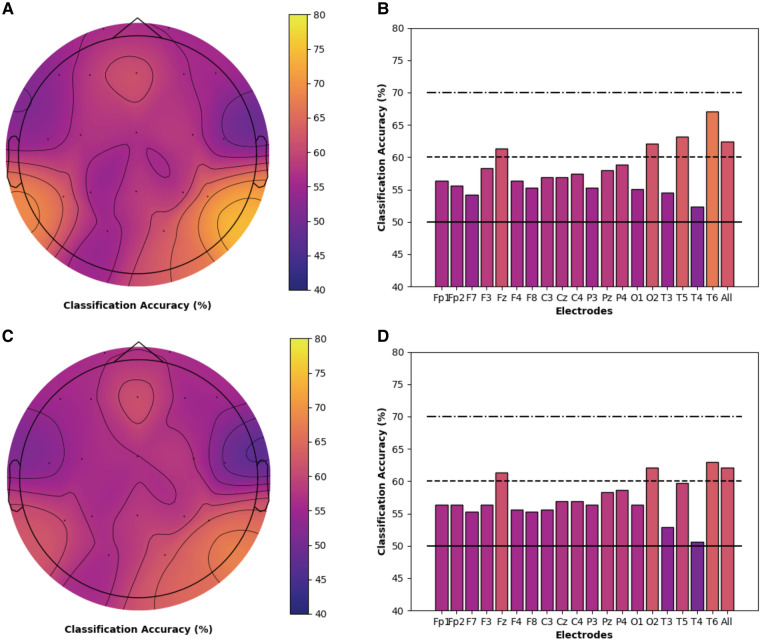
Individual electrode classification accuracy for chronic pain patients *permutation entropy features (theta frequency band)*. Pain vs. no pain classification accuracy for each individual electrode and across all electrodes for PE (theta band) features only in chronic musculoskeletal pain participants. Topographic maps and bar charts are displayed for SVM models (**A / B**) and logistic regression models (**C / D**).

**Figure 4:**
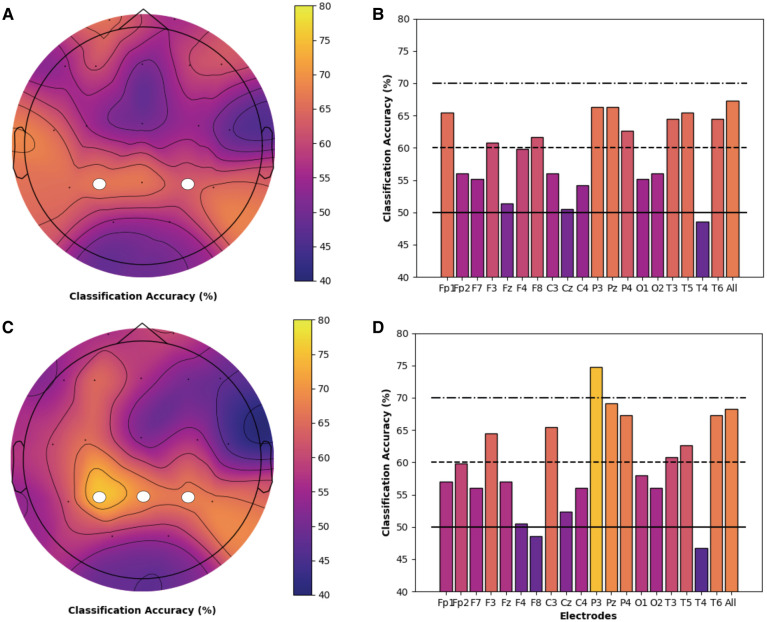
Individual electrode classification accuracy for health controls *permutation entropy features (theta frequency band)*. Pain vs. no pain classification accuracy for each individual electrode and across all electrodes for PE (theta band) features only in healthy participants. Topographic maps and bar charts are displayed for SVM models (**A / B**) and logistic regression models (**C / D**). White circles designate electrodes where models in healthy participants had significantly higher classification accuracy than chronic pain participants.

Models for both chronic pain and healthy participants highlighted the importance of PE features and the theta frequency band for the classification of cold thermal pain. Thus, we performed a secondary analysis on the classification accuracy of theta-derived PE features for each individual electrode, using the best performing SVM and logistic regression models and bootstrapping procedures described previously. Topographic maps and bar charts display the resulting classification accuracy in chronic pain (Figure 3) and healthy (Figure 4) participants. For chronic pain participants, classification accuracy ranged from 50.7% (T4) to 67.0% (T6) across individual electrodes. Higher, although more variable, performance was observed for healthy participants, with accuracies ranging from 46.7% (T4) to 74.8% (P3) across individual electrodes. Regardless of model type (SVM or logistic regression), temporal (T6), occipital (02), and frontal (Fz) electrodes had the best performance for chronic pain participants. However, PE theta models in the chronic pain group did not outperform healthy participants at any individual electrode. Parietal electrodes had the best classification accuracy for healthy participants across both models. In the logistic regression models, PE theta models in healthy participants had significantly higher classification accuracy in all parietal electrodes (Pz, P3, and P4) than chronic pain patients as evidenced by non-overlapping confidence intervals. For SVM models, only P3 and P4 electrodes outperformed the chronic pain group.

## Discussion

EEG can accurately discriminate between no-pain and pain conditions in children with chronic musculoskeletal pain and healthy youth. Classification accuracy reached 75.8% for chronic pain participants and 74.8% in healthy participants. No significant differences were observed between groups (healthy vs. chronic pain) or model types (SVM vs. logistic regression). We evaluated the most important features of model performance for each group, using the top 10% of logistic regression model weights. PE was most important EEG feature, while theta was the most significant frequency band for both groups. While features and frequencies were similar for both groups, models in adolescents with chronic musculoskeletal pain placed higher importance on features in the frontal region as opposed to healthy controls whose most significant contributors were in parietal regions.

Similar studies investigating no-pain and pain (tonic cold) conditions in healthy adult participants report classification accuracies ranging between 84.6%–93.3% (30, 31, 52–54). Due to small sample sizes (<40 participants) only one prior study used a hold-out set to validate their model, with accuracies dropping from 87.5%–93.3% on the training set to 70.9%–82.73% on the hold-out set (30). The validation accuracies from Kaur et al. (30) align with our findings; it remains a strong possibility that the remaining models may be overfitting their data, increasing their classification accuracy but limiting their generalizability. Most prior literature used spectral features to classify between conditions (30, 52, 53), which were removed from our dataset as they were significantly affected by voluntarily motor movement. It is possible that information contained in spectral analyses is important for the classification of CPT conditions, diminishing our classification accuracy; alternatively, it is possible that subtle motor activity associated with pain artificially increased classification accuracies in prior studies. Low temperature (0 °C) CPTs elicit a strong pain response, while warmer temperatures (3–7 °C) provoke pain of a lower intensity and shorter duration (55). Our CPT temperature (12 °C) was warmer than other studies and the milder stimulus may have produced a subtler neurophysiological response. However, our participants average pain rating throughout the CPT was ∼6.5, which is considered between moderate and severe on a 0–10 numeric rating scale. Furthermore, prior research found that numeric rating scales are reliability associated with clinical pain in children and numeric rating scale scores >4 are sensitive to pain requiring medication in chronic pain patients (56), thus we are confident our experiment captured a true pain response. Finally, all prior studies exclusively focused on healthy, young adult participants. Children experience vast brain maturation throughout adolescence (57) and studies in both healthy (58) and chronic pain participants (33) suggest neurophysiological differences between adults and children. The age of our participants and inclusion of chronic pain participants may also contribute to differences between our findings and prior studies with healthy adults.

PE is a quantification of information in the EEG signal (59), and our results suggest that information content changes in response to the CPT. We are the first study to investigate EEG-derived PE during a CPT, although one prior study evaluated a similar entropy-based metric. Vatankhah et al. found that no-pain and cold thermal pain classification accuracy was 14% higher using approximate entropy vs. spectral features (54). The superior classification accuracy of entropy-based features aligns with our results, as well as other studies demonstrating changes in PE outcomes in relation to pain (60, 61). Our study also aligns with prior CPT studies reporting changes in theta activity in relation to pain stimulation (27, 28, 30). Furthermore, the theta band is heavily implicated in the thalamocortical dysrhythmia (TCD) model of chronic pain. In this model, theta activity is surrounded by high frequency (beta or gamma) activity (62), which disconnects normal circuit function. A thalamocortical column-specific decrease in information processing is created, which is thought to ultimately resulting in the oscillatory mechanism that propagates the painful condition (63, 64). Although our results do not provide direct evidence of the TCD model of chronic pain, the importance of PE features and theta frequency activity point to the important role of information processing in pain.

Prior studies in healthy participants highlight both frontal (27, 28, 30) and parietal (28, 30) responses to simulated pain during a CPT. Additional studies also provide support that chronic pain participants have alterations in frontal (23) and parietal (64) areas compared to healthy controls at rest. Pain is relayed to the thalamus from the spinal cord, where it is splits into two distinct pain systems: 1) the lateral pain system, which includes the somatosensory cortex, involved in the perception of pain (sensory and discrimination); and 2) the medial pain system, including the prefrontal cortex, which encodes the emotional aspects of pain (affect and motivation) (65, 66). The majority of studies using tonic experimental pain conditions show an activation in the lateral pain system, but less than 25% of studies in chronic pain participants show changes to the somatosensory cortex (65). In fact, chronic pain participants often show increased activation in medial pain system structures and decreased activation in the somatosensory cortex compared to healthy controls at rest, which couples with observations that chronic pain leads to decreased sensory processing and increased affective processing (67, 68). Our results agree with this literature and generally suggest that adolescents with chronic musculoskeletal pain predominantly contextualize cold thermal pain through affective systems, while healthy controls have a sensory-based experience of tonic cold pain.

### Strengths

The inclusion of a large cohort (*n* = 121) of pediatric chronic pain participants is a major strength of the current study, as prior work has primarily evaluated small samples of healthy adults. Our study design allowed for the development of models specific to chronic pain participants, which differed from healthy children in the locations of the most discriminatory EEG features. The chronic pain group was highly variable in relation to their specific diagnosis, location, and frequency of pain. Our heterogeneous sample is representative of the diverse presentations of chronic pain patients seeking clinical care and increases the generalizability of our model to various pain phenotypes. Prior literature focused predominantly on spectral EEG features, a limitation that has been noted previously (32). Our methods included spectral, entropy, functional connectivity, and graph theory feature types, many of which were previously unexplored in pediatric chronic pain participants. The inclusion of PE features was particularly prudent, as these features best classified tonic cold pain and represent an important contribution to the pain literature. Finally, we systematically evaluated and removed features that were affected by voluntary motor movement. Subjective pain ratings are collected serially through thermal QST and typically require verbal indication or movement (pointing to a number, moving a sliding scale, etc.). Thus, the potential for motor-based artefact entering the EEG signal is high. The removal of such features provides confidence that our findings result from the neurophysiological changes underlying cold pain, not motor activity. Finally, our classification is not reliant upon subjective pain reports. Beyond providing objective evidence to clinicians, this approach can be particularly beneficial in younger children who do not have the language or cognitive capacity to adequately verbalize their pain or in children with disabilities who may be nonverbal.

### Limitations

The recruitment of healthy controls was largely affected by the COVID-19 pandemic. Although our healthy participant sample (*n* = 39) is very comparable to prior studies, the imbalance in group size prevented models directly comparing chronic pain to healthy children at baseline and during the CPT. We intentionally selected our approach to focus on objective, physiological outcomes to evaluate metrics that could be broadly generalizable to young and/or populations that may not be able to complete common self-reported pain scales. However, as pain varies throughout the CPT, modeling subjective pain (e.g. numeric rating scale values during the CPT) in addition to classifying condition (e.g. baseline vs. CPT) would have strengthened our results. However, subjective pain ratings (15sec) were not collected on the same time scale as EEG epochs (10sec). Therefore, subjective pain ratings were not available for every EEG epoch and could not be modeled with our data. A significantly higher proportion of females was present in the chronic pain group, which aligns with prior literature suggesting chronic pain is more prevalent in girls (2, 69). However, as EEG is affected by both age (70) and sex (71), the relatively wide age range (10–18 years) during an important maturation period and imbalance between sexes in the chronic pain group may have decreased model performance. We constrained our ML approaches to common, supervised learning methods. Future studies should evaluate other approaches, including unsupervised learning. Regardless of model type, more extensive external validation (replicating results on more participants from different geographical regions and different EEG systems, etc.) is needed before translation to clinical settings. Denser electrodes arrays limit clinical translation, but provide opportunities for sophisticated analysis techniques (e.g., source localization, local graph theory metrics, etc.) that provide more insight into the specific brain areas and networks underlying the perception of pain. Finally, we selected a tonic thermal stimulus as this more closely mimics clinical pain and allows for sufficiently long EEG recording periods. However, other forms of pain stimulation (e.g., pin prick, pressure) are also used in comprehensive clinical pain assessments and should be evaluated in conjunction with EEG recordings when feasible.

### Clinical implications

An objective, physiological biomarker of pediatric chronic pain has the potential to improve clinical care and overall quality of life. Children may not have the developmental abilities or language capacity to clearly and thoroughly describe their pain to clinicians; this is particularly true for more vulnerable populations (e.g. non-verbal children). Even when they can adequately verbalize their pain, children often report that their pain was “disbelieved” or “dismissed” by their physician (72). Thus, a biomarker could provide objective evidence that a child is experiencing pain, which may facilitate earlier enrollment into treatments capable of effectively managing the child's pain. PE features, which can be generated from a single EEG electrode, were determined to be the most important feature for both groups. This increases the potential for clinical translation, as a single-metric, single-electrode biomarker greatly reduces the burden, time, and cost associated with EEG. In healthy participants, theta PE at the P3 electrode produced identical classification accuracy to all EEG features and frequency bands, although the highest single-electrode accuracy in chronic pain participants was ∼7% lower than the full model. Still, our results highlight the potential for PE features as an objective marker of pain in the absence of self-report. Furthermore, PE features are robust to noise sources and artefacts (73), which is critical in busy recording environment such as clinical settings. Equivalent results were obtained using SVM and logistic regression models, which can have important implications for translation. Some clinicians can be skeptical of ML approaches, as the transformations used to generate high discrimination accuracy often comes at the expense of understanding the specific features, patterns, or neurophysiological responses underlying model performance (74). Thus, the ability to replicate findings using an inferential statistics approach speaks to the consistent performance of model and may increase uptake in clinical settings, as clinicians can extract the specific neural correlates associated with a subjective pain response.

## Conclusions

EEG features can accurately and significantly discriminate between no-pain and tonic cold pain conditions in adolescents with chronic musculoskeletal pain and healthy controls. Final classification accuracy ranged from 72.0%–75.8%, with no significant differences observed between groups (healthy vs. chronic pain) or models (SVM vs. logistic regression). PE features in the theta frequency bands had the best discrimination for both chronic pain and healthy participants, although the specific regions of importance differed between groups. Overall, our results demonstrate the feasibility of accurately detecting subjective pain experience from electrophysiological data and represent the first step towards the development of a point-of-care system to detect pain in the absence of self-report.

## Data Availability

The raw data supporting the conclusions of this article will be made available by the authors, without undue reservation.
